# Use of Defibrotide to help prevent post-transplant endothelial injury in a genetically predisposed infant with metachromatic leukodystrophy undergoing hematopoietic stem cell gene therapy

**DOI:** 10.1038/s41409-017-0085-1

**Published:** 2018-01-29

**Authors:** Valeria Calbi, Francesca Fumagalli, Giulia Consiglieri, Rachele Penati, Serena Acquati, Daniela Redaelli, Vanessa Attanasio, Facchini Marcella, Maria Pia Cicalese, Maddalena Migliavacca, Federica Barzaghi, Francesca Ferrua, Andrea Assanelli, Paolo Silvani, Matteo Zoccolillo, Giovanna Chidini, Robert Chiesa, Ruchi Arora, Francesca Ciotti, Marina Sarzana, Gigliola Antonioli, Cristina Baldoli, Francesco Morena, Sabata Martino, Gian Luigi Ardissino, Maria Grazia Natali Sora, Luigi Naldini, Fabio Ciceri, Alessandro Aiuti, Maria Ester Bernardo

**Affiliations:** 1 San Raffaele Telethon Institute for Gene Therapy (SR-TIGET), IRCCS San Raffaele Scientific Institute, Milan, Italy; 2 Pediatric Immunohematology Unit and Stem Cell Program, IRCCS San Raffaele Scientific Institute, Milan, Italy; 3 Neurology Department, IRCCS San Raffaele Scientific Institute, Milan, Italy; 4 Vita-Salute San Raffaele University, Milan, Italy; 5 Unit of Hematology and Bone Marrow Transplantation, IRCCS San Raffaele Scientific Institute, Milan, Italy; 6 Department of Anesthesia and Critical Care, IRCCS San Raffaele Scientific Institute, Milan, Italy; 7 Pediatric Intensive Care Unit, Department of Anesthesia and Critical Care, Fondazione IRCCS Ca’ Granda, Ospedale Maggiore Policlinico, Milan, Italy; 8 Bone Marrow Transplantation Unit, Great Ormond Street Hospital, London, UK; 9 Department of Paediatrics, Norfolk and Norwich University Hospital, Norwich, UK; 10 Neuroradiology Unit, IRCCS San Raffaele Scientific Institute, Milan, Italy; 11 Department of Chemistry, Biology and Biotechnologies, University of Perugia, Perugia, Italy; 12 HUS Center, Fondazione IRCCS Ca’ Granda Ospedale Maggiore Policlinico, Milan, Italy

Metachromatic Leukodystrophy (MLD) is a fatal demyelinating lysosomal storage disease with no approved treatment; it is caused by mutation in arylsulfatase A (*ARSA*) gene that results in accumulation of sulphatides in neural and glial cells. Patients with symptom onset before 30 months of age are defined as late-infantile (LI) form and have a severe clinical course characterized by a very rapid neurological deterioration. While allogeneic hematopoietic stem cell transplantation (HSCT) shows limited benefit in early onset forms of MLD, preliminary results of hematopoietic stem cell-gene therapy (HSC-GT) show evidence of safety and clinical benefit in pre-symptomatic LI patients [1].

Injury of vascular endothelium observed within the first 30–60 days after HSCT leads to several complications including Hepatic Veno-Occlusive Disease (VOD) and Thrombotic Microangiopathy (TMA). VOD is characterized by platelet transfusion refractoriness, weight gain, fluid retention, ascites, painful hepatomegaly, and eventually multi-organ dysfunction with high mortality rate [2, 3]. Well-established risk factors for VOD are: young age, pre-existing liver damage and use of busulfan-based myeloablative conditioning regimens [4]. Moreover, there is an increased risk of VOD in some inherited metabolic disorders [5] and in patients with genetic polymorphisms of various enzymes [6]. Defibrotide (DF) is approved for the treatment of VOD and is able to reduce endothelial activation, protect endothelial cells and improve thrombo-fibrinolytic balance [7]. TMA is characterized by endothelial activation and microvascular thrombosis; known clinical risk factors for TMA include calcineurin inhibitor use, busulfan chemotherapy, allogeneic HSCT, Graft-versus-Host Disease and previous VOD. Post-transplant TMA can present as atypical Hemolytic uremic syndrome (aHUS) with occurrence of microangiopathic hemolysis, platelet consumption and acute kidney injury [8]. This latter syndrome is often caused by acquired or genetic dysregulation of the complement system, most commonly gene mutations in complement factor H (CFH), factor I (CFI), factor B (CFB), complement component 3 (C3), membrane cofactor protein (MCP) or acquired anti-CFH antibodies [9]. The anti-C5 monoclonal antibody Eculizumab has been successfully utilized for aHUS treatment, being able to block the complement cascade [10].

Here we report the case of two monozygous twins affected by LI-MLD undergoing HSC-GT with CD34^+^ cells transduced with a lentiviral vector encoding for the ARSA gene who were subsequently discovered to also harbour a MCP gene mutation; while the first transplanted twin developed severe VOD and TMA, DF prophylaxis and reduction of busulfan regimen were associated with absence of these complications in the second twin.

Twins were diagnosed with MLD at 3 months of age by genetic testing (heterozygous 465 + 1 G > A and c.240dup mutations in the *ARSA* gene) and low ARSA activity following disease onset in an older sibling. They received HSC-GT at the pre-symptomatic stage; the dose of transduced CD34^+^ cells infused was similar in the 2 patients (18.2 × 10^6^/kg for Patient 1 (Pt1); 14.1 × 10^6^/kg for Patient 2 (Pt2) (Table 1).

**Table 1:** Disease and transplant characteristics of Patient 1 and 2

Pre-transplant characteristics						Conditioning regimen			
	Age at MLD Diagnosis	Age at Treatment	ARSA gene Mutation analysis	ARSA Activity (n.v. 22–103)	MLD type	Bu AUC	Infused cell dose	Engraftment	
								*N* > 0.5 × 10^9^/L	PLT > 50 × 10^9^/L
**Pt 1**	3 months	8 months	465 + 1 G > A c.240dup	1.4 nmol/h/mg	LI pre-symptomatic	84.9 mg h/L	18.2 × 10^6^ CD34 + /kg	day +39	day +109
**Pt 2**	3 months	9 months	465 + 1 G > A c.240dup	1.4 nmol/h/mg	LI pre-symptomatic	63.4 mg h/L	14.1 × 10^6^ CD34 + /kg	day +43	day +81

*Pt* patient, *ARSA* arylsulfatase A, *MLD* metachromatic leukodystrophy, *LI* late-infantile, *Bu* busulfan, *AUC* area under the curve, *N* neutrophil, *PLT* platelet.

At 8 months of age, Pt1 received myeloablative conditioning with i.v. busulfan with target AUC of 85 mg h/L (actual extrapolated exposure 84.9 mg h/L). On day (d) + 18 after HSC-GT, he developed severe VOD, diagnosed according to modified Seattle criteria [11], and DF (25 mg/kg/day) was initiated the same day. Rapid and marked increase of liver enzymes (ALT peak value was 617 UI/L) and abundant ascites that required ultrasound-guided drainage followed the start of DF; subsequently the patient progressively improved including remission of platelet transfusion refractoriness on day +24 and remission of all signs and symptoms of VOD day +30. From day +32 he showed recurrence of refractoriness to platelet transfusions with signs of hemolysis, marked proteinuria with C3 reduction and schistocytes on peripheral blood smear, pointing to a diagnosis of TMA. Direct Coombs test and anti-platelet antibodies were positive. Eculizumab 300 mg i.v. weekly was started (Fig. 1). The child became drowsy and developed respiratory distress with need of C-PAP and pediatric intensive care unit admission. Anti-CFH antibodies resulted strongly positive and this was confirmed also in pre-GT plasma samples. Genetic analyses revealed a heterozygous deletion of CFHR3-R1 (irrelevant for anti-CFH antibodies) and Ala353Val mutation of MCP gene, which has been associated with inadequate control of complement activation [12]. Due to persistence of anti-platelet antibodies and anti-CFH antibodies, a course of Rituximab (375 mg/m^2^; 4 weekly doses) was administered. The patient’s clinical condition eventually improved, although he showed prolonged anaemia and thrombocytopenia (in the absence of active haemolytic process) with secondary gastrointestinal bleeding. He required unmanipulated autologous back-up bone marrow infusion to boost hematological recovery on day +66. At the latest follow-up he showed good hematopoietic and immune reconstitution, no signs of renal damage, albeit with neurodevelopmental delay; at the age of 30 months he can walk with support.

**Fig. 1:**
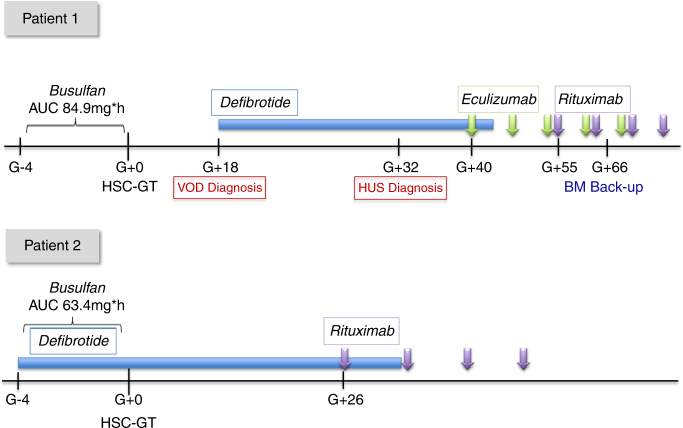
Treatment schedule of the 2 patients undergoing HSC-GT. Patient 1: Diagnosis of VOD on day +18 was based on the modified Seattle criteria: hepatomegaly and increased body weight >2%;. Reduced urine output and refractoriness to PLT transfusions preceded hepatomegaly and ascites; this confirms the importance of EBMT revised pediatric diagnostic criteria for VOD recently published (Corbacioglu S. et al.). In this case, the increase of D-dimer was documented very early in the course of VOD, while increase of hepatic necrosis enzymes (peak values ALT 617 UI/L, AST 1040 UI/L, LDH 759 UI/L), bilirubin (peak value of total bilirubin 2.67 mg/dL, direct 1.48 mg/dL) and need of ascetic fluid drainage followed the start of Defibrotide. Defibrotide (Defitelio) 25 mg/kg/day i.v. QID was given from d + 18 to d + 41 resulting in remission of all signs and symptoms of VOD. Diagnosis of TMA on d + 32 was based on relapse of refractoriness to platelet transfusions, increase in indirect bilirubin values (up to 3.02 mg/dl), reduction of haptoglobin, presence of schistocytes 2–3% in peripheral blood smear and urine protein/creatinine ratio (peak value >10). Anti-CHF antibodies were positive (1371 UI/ml), accompanied by a reduction of complement factors (C3 0.77 g/L, C4 0.07 g/L). Eculizumab (Soliris) 300 mg i.v. weekly for 4 doses was administered from d + 40, followed by 2 maintenance doses (300 mg i.v.) every 2 weeks. On d + 55, Rituximab 375 mg/m^2^ i.v. for 4 weekly doses was started due to persistent presence of anti-CFH and anti-platelet antibodies with low PLT counts. On d + 66, due to delayed hematological recovery, back-up reinfusion was performed. Patient 2: received prophylactic Defibrotide 25 mg/kg/day i.v. QID from d-4 before HSC-GT to d + 30. Rituximab 375 mg/m^2^ was administered from d + 26 for 4 doses, due to presence of anti-CHF and anti-PLT antibodies. He did not develop signs of VOD or TMA

Considering the onset of severe VOD and TMA in his monozygous twin, DF prophylaxis was instituted in Pt2 from day −4 to day +30. The busulfan regimen administered was myeloablative, but with a reduced target AUC (67.2 mg*h/L). Actual extrapolated exposure was 63.4 mg*h/L. The child did not develop signs of VOD, complement activation or renal function impairment. However, anti-CFH antibodies resulted positive before HSC-GT and at day +12, while anti-platelet antibodies resulted positive at day +14; therefore, a course of 4-weekly Rituximab doses was administered (Fig. 1). The patient showed slow hematological recovery (Table 1) in the absence of clinical complications. He is currently 20 months post-HSC-GT with full hematological recovery, persistent engraftment of gene corrected cells, no signs of microangiopathy and progressive acquisition of motor and cognitive developmental milestones. In both patients proportion of gene corrected cells and ARSA activity was in line with previous patients treated with HSC-GT but markedly higher in pt 2.

These LI-MLD monozygous twins harboured two mutations in complement regulator genes (CRG), one known missense mutation in MCP and a heterozygous deletion in CFHR3-R1, as well as the presence of anti-platelet and anti-CFH antibodies. Whole genome sequencing identified a homozygous variant polymorphism in *Heparanase* gene (HPSE; rs4364254 C > T) associated with higher risk of VOD [13], and a second homozygous variant in Glutathione transferase-A2 gene (GST-A2; rs2180314, p.Thr112Ser), a haplotype linked to decreased busulfan clearance and higher bilirubin levels [14]. Studies have shown that phenotypes and clinical manifestations in patients with mutations in CRG depend on environmental triggers. In particular, MCP mutations are characterized by a high rate of incomplete penetrance [12], appearing to be a predisposing factor for TMA development, rather than causative of TMA. Anti-CFH antibodies are detected at higher prevalence in patients who develop aHUS than in healthy subjects, suggesting that immune/inflammatory dysregulation can predispose to development of sporadic aHUS (Dragon-Durey et al., 2010). Prognosis varies with each phenotype and among gene abnormalities; those involving MCP are associated with the best prognosis [12].

Mutations in CRG and anti-CFH antibodies have not been evaluated as possible risk factors for VOD. However, clinical features of VOD, such as refractoriness to platelet transfusion and microvessel thrombosis, may suggest that VOD and TMA are a continuous spectrum of a common pathogenic process that leads to inflammation, endothelial activation and microvascular thrombosis with secondary organ damage.

Pt1 experienced severe VOD, successfully treated with DF, and TMA, which resolved with Eculizumab and Rituximab administration. Considering these severe complications when planning treatment of his monozygous brother, we decided to modify environmental triggers, which may have been responsible for VOD and TMA, as the penetrance in aHUS is estimated to be around 50% [6, 9, 15]. DF prophylaxis was instituted and myeloablative busulfan regimen was slightly modified by decreasing systemic exposure; this was associated with the absence of both VOD and TMA in a high-risk patient.

This unique case–control study in monozygous twins contributes to our understanding of the pathophysiology of endothelial damage after HSCT; we speculate that endothelial protection conferred by DF in a child genetically predisposed to develop microangiopathy might have contributed to prevention of secondary organ damage. Adjustment of busulfan exposure to a lower, although myeloablative, AUC may have also played a role in reducing the degree of endothelial injury. This case report also indicates that in selected situations, extensive evaluation of molecular polymorphisms by whole genome sequencing can identify genetically predisposed high-risk patients and guide appropriate prophylactic measures. Finally, it underlines that prompt diagnosis and proper prophylaxis and treatment of VOD and TMA may help overcome genetic predisposing factors and prevent severe complications and multiple-organ damage.

## References

[1] Sessa M, Lorioli L, Fumagalli F, Acquati S, Redaelli D, Baldoli C (2016). Lentiviral haemopoietic stem-cell gene therapy in early-onset metachromatic leukodystrophy: an ad-hoc analysis of a non-randomised, open-label, phase 1/2 trial. Lancet.

[2] Corbacioglu S, Cesaro S, Faraci M, Valteau-Couanet D, Gruhn B, Rovelli A (2012). Defibrotide for prophylaxis of hepatic veno-occlusive disease in paediatric haemopoietic stem-cell transplantation: an open-label, phase 3, randomised controlled trial. Lancet.

[3] Corbacioglu S, Carreras E, Ansari M, Balduzzi A, Cesaro S, Dalle JH, et al. Diagnosis and severity criteria for sinusoidal obstruction syndrome/veno-occlusive disease in pediatric patients: a new classification from the European society for blood and marrow transplantation. Bone Marrow Transplant. 2017. 10.1038/bmt.2017.161. 10.1038/bmt.2017.161 PMC5803572 28759025

[4] Hopps SA, Borders EB, Hagemann TM (2016). Prophylaxis and treatment recommendations for sinusoidal obstruction syndrome in adult and pediatric patients undergoing hematopoietic stem cell transplant: a review of the literature. J Oncol Pharm Pract.

[5] Corbacioglu S, Hönig M, Lahr G, Stöhr S, Berry G, Friedrich W, Schulz AS (2006). Stem cell transplantation in children with infantile osteopetrosis is associated with a high incidence of VOD, which could be prevented with defibrotide. Bone Marrow Transplant.

[6] Mohty M, Malard F, Abecassis M, Aerts E, Alaskar AS, Aljurf M (2016). Revised diagnosis and severity criteria for sinusoidal obstruction syndrome/veno-occlusive disease in adult patients: a new classification from the European Society for Blood and Marrow Transplantation. Bone Marrow Transplant.

[7] Richardson PG, Riches ML, Kernan NA, Brochstein JA, Mineishi S, Termuhlen AM (2016). Phase 3 trial of defibrotide for the treatment of severe veno-occlusive disease and multi-organ failure. Blood.

[8] Obut F, Kasinath V, Abdi R (2016). Post-bone marrow transplant thrombotic microangiopathy. Bone Marrow Transplant.

[9] Geerdink LM, Westra D, Van Wijk JA, Dorresteijn EM, Lilien MR, Davin JC (2012). Atypical hemolytic uremic syndrome in children: complement mutations and clinical characteristics. Pediatr Nephrol.

[10] Wong EK, Goodship TH, Kavanagh D (2013). Complement therapy in atypical haemolytic uraemic syndrome (aHUS). Mol Immunol.

[11] McDonald GB, Sharma P, Matthews DE, Shulman HM, Thomas ED (1984). Venocclusive disease of the liver after bone marrow transplantation: diagnosis, incidence, and predisposing factors. Hepatology.

[12] Liszewski MK, Atkinson JP (2015). Complement regulator CD46: genetic variants and disease associations. Hum Genom.

[13] Seifert C, Wittig S, Arndt C, Gruhn B (2015). Heparanase polymorphisms: influence on incidence of hepatic sinusoidal obstruction syndrome in children undergoing allogeneic hematopoietic stem cell transplantation. J Cancer Res Clin Oncol.

[14] Bonifazi F, Storci G, Bandini G, Marasco E, Dan E, Zani E (2014). Glutathione transferase-A2 S112T polymorphism predicts survival, transplant-related mortality, busulfan and bilirubin blood levels after allogeneic stem cell transplantation. Haematologica.

[15] Dragon-Durey MA, Blanc C, Garnier A, Hofer J, Sethi SK, Zimmerhackl LB (2010). Anti-factor H autoantibody-associated hemolytic uremic syndrome: review of literature of the autoimmune form of HUS. Semin Thromb Hemost.

